# An Instant Relationship Between Hyponatremia, Geriatric Syndromes, and Drugs in Older Adults: A Cross-Sectional Analysis from a Single Geriatric Clinic

**DOI:** 10.3390/diagnostics15060744

**Published:** 2025-03-16

**Authors:** Ali Ekrem Aydin, Esra Ates Bulut, Suleyman Emre Kocyigit, Fatma Sena Dost, Feyza Mutlay, Kubra Altunkalem Seydi, Fethiye Esenkaya, Ahmet Turan Isik

**Affiliations:** 1 Department of Geriatric Medicine, School of Medicine, Ondokuz Mayis University, 55139 Samsun, Türkiye; 2 Department of Geriatric Medicine, Adana City Training and Research Hospital, University of Health Sciences, 01370 Adana, Türkiye; esraates@yahoo.com; 3 Department of Geriatric Medicine, School of Medicine, Balikesir University, 10145 Balikesir, Türkiye; suleymanemrekocyigit@gmail.com; 4 Unit for Brain Aging and Dementia, Department of Geriatric Medicine, School of Medicine, Dokuz Eylul University, 35340 Izmir, Türkiye; sena_dost@hotmail.com (F.S.D.); atisik@yahoo.com (A.T.I.); 5 Division of Geriatric Medicine, Van Training and Research Hospital, 65300 Van, Türkiye; feyzamutlay@hotmail.com; 6 Division of Geriatric Medicine, Sultan 1. Murat State Hospital, 22030 Edirne, Türkiye; kubraltnklm@hotmail.com; 7 Department of Genetic Medicine, Izmir City Training and Research Hospital, 35540 Izmir, Türkiye; fethiyeocakdan@gmail.com

**Keywords:** hyponatremia, geriatric syndromes, drugs, older adult, trazodone

## Abstract

**Background**: Hyponatremia is a common electrolyte disorder in older adults that can lead to poor clinical outcomes and increased mortality. This study aims to evaluate the interrelationship between hyponatremia and geriatric syndromes and drugs in older adults. **Methods**: This study included 1100 elderly patients admitted to a geriatric clinic. Patient records were used to obtain demographic information, comorbidities, geriatric syndromes, medications, laboratory results, and comprehensive geriatric assessment parameters. **Results**: The prevalence of hyponatremia was 23.9% in this study (mean age ± SD was 75.59 ± 8.13 years). The frequency of polypharmacy, dementia, falls, malnutrition and risk of malnutrition, frailty, probable sarcopenia, hypertension, cerebrovascular disease, and congestive heart failure was higher, and patients were older in the hyponatremia group (*p* < 0.05) than in the normonatremia group. After the adjustment of covariates, hyponatremia was shown to be related to drugs including escitalopram (odds ratio [OR]: 1.82, 95% confidence interval [CI]: 1.20–2.76), trazodone (OR: 2.27, 95% CI: 1.26–4.10), renin angiotensin aldosterone system (RAAS) inhibitors (OR: 1.71, 95% CI: 1.18–2.47), hydrochlorothiazide (OR: 1.83, 95% CI: 1.28–2.62), and opioids (OR: 4.46, 95% CI: 1.24–16.02) (*p* < 0.05). Polypharmacy, falls, and malnutrition with risk of malnutrition were still significantly associated with increased hyponatremia risk even after adjustment for age, sex, and comorbidity burden (*p* < 0.05). **Conclusions**: Hyponatremia seems to be associated with certain geriatric syndromes, as well as the use of some antidepressants and cardiovascular drugs in older adults. Malnourished older adults taking RAAS inhibitors, diuretics, opioids, and antidepressants may be at a higher risk of developing hyponatremia. They should be closely monitored, especially if they are taking multiple medications.

## 1. Introduction

Hyponatremia, the most common electrolyte disorder leading to poor clinical outcomes and increased mortality in older patients, is defined as a serum sodium concentration below 135 mmol/L [1]; it is usually mild (between 130 and 135 mmol/L) and chronic (developing in >48 h) [2]. The prevalence has varied from nearly 7% in community-dwelling older adults to 18% in nursing homes [3], whereas it may reach 40% in intensive care settings [4]. Hyponatremia may lead to many unfavorable severe health consequences, such as an increased risk of hospitalization, longer hospital stays, and higher mortality rates [5]. Furthermore, mounting evidence suggests that chronic hyponatremia, even if it is asymptomatic, may be associated with fatigue, cognitive impairment, impaired balance, falls, bone demineralization, fractures, and frailty [2,3,6]. Therefore, it is essential to clarify the factors that cause hyponatremia. Age-related changes, comorbidities, and medications, as well as aging, an independent risk factor, render older adults vulnerable to hyponatremia [7]. These changes include impaired urine concentration capacity, decreased total body water, and changed antidiuretic hormone (ADH) sensitivity, whereas the comorbidities include endocrinopathies, liver disease, and heart failure. Furthermore, as in other medical conditions in geriatric practice, medications, especially diuretics, are of particular importance in the development of hyponatremia. Among them, thiazide diuretics, by increasing the loss of sodium, and certain drugs, such as antidepressants, antipsychotics, anticonvulsants, and anti-cancer medications, by leading to syndrome of inappropriate antidiuretic hormone secretion (SIADH), may cause chronic hyponatremia. In addition, SIADH may also be due to pulmonary diseases (like carcinoma, pneumonia, etc.), intracranial space-occupying lesions, infections, and other cancers [8,9].

Considering the possible consequences of hyponatremia, whether acute or chronic, and the nature and diversity of geriatric syndromes, there may be a bidirectional relationship between both conditions in older adults. For example, most geriatric syndromes, such as malnutrition, dementia, polypharmacy, and frailty, may directly or indirectly result in hyponatremia, deepen its severity, or make its treatment difficult. In contrast, hyponatremia is a precipitating factor for delirium and may contribute to falling by leading to headaches and confusion caused by hyponatremia. The relationship between geriatric syndromes, drugs, and systemic diseases is complex. It is difficult to explain the pathophysiological mechanisms since older adults constitute heterogeneous patients, and the number of studies investigating geriatric syndromes of hyponatremia in older adults is limited [2,10]. In addition, hyponatremia, a reversible cause of severe unwanted health conditions, can be effectively corrected when the underlying reason and coexisting situations are revealed.

Therefore, this study aimed to examine the geriatric syndromes, comorbidities, and drugs associated with hyponatremia in older adults.

## 2. Patients and Methods

### 2.1. Study Population and Study Design

We conducted a retrospective, cross-sectional, hospital record-based, single-center observational study on 1100 older adults admitted to the geriatric clinic between January 2013 and December 2018. This study documented the participants’ demographic features, comorbidities, geriatric syndromes, drugs, laboratory findings, and Basic and Instrumental Activities of Daily Living (ADL) scores.

#### 2.1.1. Inclusion Criteria

Patients over 60 years of age who were admitted to the Department of Geriatrics for any health reason and did not meet the exclusion criteria were included in this study.

#### 2.1.2. Exclusion Criteria

**Figure 1:**
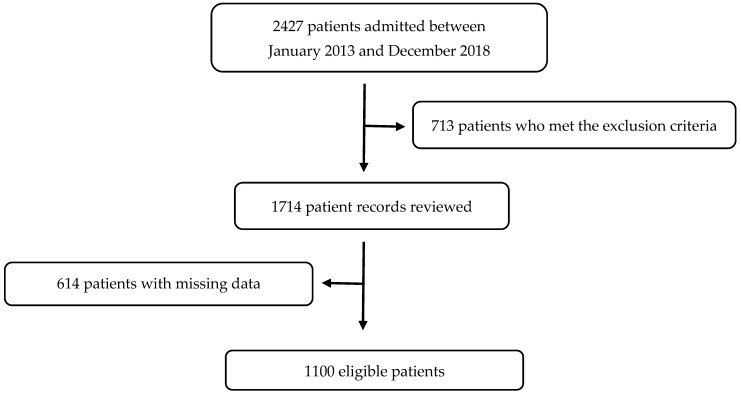
Flowchart of this study.

Patients with septic shock, acute adrenal deficiency, The New York Heart Association Functional Classification class 3 or 4 heart failure, pulmonary edema, hypotensive or septic shock, coma, gastrointestinal bleeding, or severe prior stroke or myocardial infarction in the past seven days, as well as those under 60 years of age, were excluded. Following the exclusion criteria, 1100 patients were enrolled in this study; the flow chart of this study is presented in Figure 1.

### 2.2. Definition of Hyponatremia

In the literature, the upper limit of hyponatremia varies across studies. Hyponatremia was considered as plasma sodium levels below 135 mmol/L in this study [11]. The sodium and glucose levels were recorded, and serum sodium was corrected for serum glucose levels using a correction factor of 1.6 mmol/L decreases in serum sodium concentration per 100 mg/dL serum glucose increment [12]. All participants were divided into two groups: hyponatremia and normonatremia.

### 2.3. Patients’ Characteristics

Demographic characteristics were recorded. The patients’ records were assessed for comorbidities. Routine biochemical laboratory data of the patients were obtained from hospital laboratory records. All these biochemical tests were carried out on a Diagnostic Modular Systems autoanalyzer (E170 and P800 Diagnostic modular systems; Roche, Basel, Switzerland). In addition, for each patient, scores of the Basic and Instrumental Activities of Daily Livings (ADLs), Yesavage Geriatric Depression Scale (YGDS), Performance-Oriented Mobility Assessment (POMA), Mini-Mental State Examination (MMSE) indexes, timed up-and-go duration, and drug lists were also recorded as a part of comprehensive geriatric assessment from the patient files [13,14]. The Charlson Comorbidity Index (CCI) score was calculated based on the International Classification of Diseases diagnosis codes with 19 category weights from 1 to 6 [15]. Considering the drugs associated with hyponatremia in the literature, those used by the patients and their daily doses were as follows: antidepressants (escitalopram 10–20 mg, citalopram 20–40 mg, sertraline 50–100 mg, duloxetine 30–60 mg, venlafaxine 75–150 g mirtazapine 15–30 mg, paroxetine 10–20 mg, trazodone 25–100 mg), antipsychotics (risperidone 1–2 mg, quetiapine 12.5–50 mg, olanzapine 5–10 mg, aripiprazole 5 mg), proton pump inhibitors (PPIs) (pantoprazole 40 mg, lansoprazole 30 mg, esomeprazole 40 mg, rabeprazole 10–20 mg), renin–angiotensin–aldosterone system (RAAS) inhibitors (olmesartan 10–40 mg, valsartan 80–320 mg, candesartan 8–32 mg, irbesartan 75–300 mg, telmisartan 80 mg, ramipril 2.5–10 mg, perindopril 4–10 mg, zofenopril 15–30 mg, enalapril 20 mg, trandolapril 2–4 mg, lisinopril 20 mg), diuretics (hydrochlorothiazide 12.5–25 mg, indapamide 1.25–2.5 mg, furosemide 20–80 mg), opioids (tramadol 50–150 mg, fentanyl transdermal patch 12–25 mcg/h), and NSAIDs (diclofenac 25–50 mg, dexketoprofen 25 mg, flurbiprofen 100 mg, naproxen 550 mg, etodolac 400 mg, indomethacin 25–50 mg, meloxicam 15 mg).

### 2.4. Geriatric Syndromes

The following eight geriatric syndromes were evaluated: falls, urinary incontinence, dementia, depression, frailty, polypharmacy, malnutrition, and sarcopenia. Falls were considered positive if the patient had experienced more than one fall within the last year without tripping. Urinary incontinence was defined as having involuntary urinary leakage in the last three months, except for urinary tract infection [16]. Dementia and depression were diagnosed based on the Diagnostic and Statistical Manual of Mental Disorders, Fifth Edition (DSM-V) criteria. Frailty was evaluated using the Fried Physical Frailty Scale [17]. The presence of three or more of the five criteria (exhaustion, slow walking speed, low muscle strength, weight loss, low physical activity) was identified as frailty. Patients using five or more concomitant drugs were considered to be polypharmacy [18]. Malnutrition and malnutrition risk were screened with the Mini-Nutritional Assessment Short Form (≤11) [13]. Probable sarcopenia was diagnosed according to the revised European Working Group on Sarcopenia in Older People (EWGSOP). Patients with low handgrip strength were considered to have probable sarcopenia according to the Turkish cut-off values [19]. Handgrip strength was measured three times for each hand using the Jamar handheld hydraulic dynamometer (Sammons Preston, Inc., Bolingbrook, IL, USA).

### 2.5. Statistical Analysis

First, the participants were divided into two groups: hyponatremia and normonatremia. Continuous variables were assessed as means and standard deviations and were evaluated by the Kolmogorov–Smirnov test for normal distribution. As all variables were not normally distributed, the continuous variables were evaluated using the Mann—Whitney U test. Chi-square and Fisher’s exact Chi-square tests were used to evaluate the differences between the categorical variables. Binary logistic regression analysis was performed to assess the factors associated with hyponatremia. Covariates [age, sex, drug number (except for polypharmacy), comorbidity burden, glucose, albumin, and estimated glomerular filtration rate (eGFR)] were also adjusted in a second model. All statistical analyses were carried out using SPSS 25.0 (SPSS Inc., Chicago, IL, USA). A probability of <0.05 was considered significant.

## 3. Results

**Figure 2:**
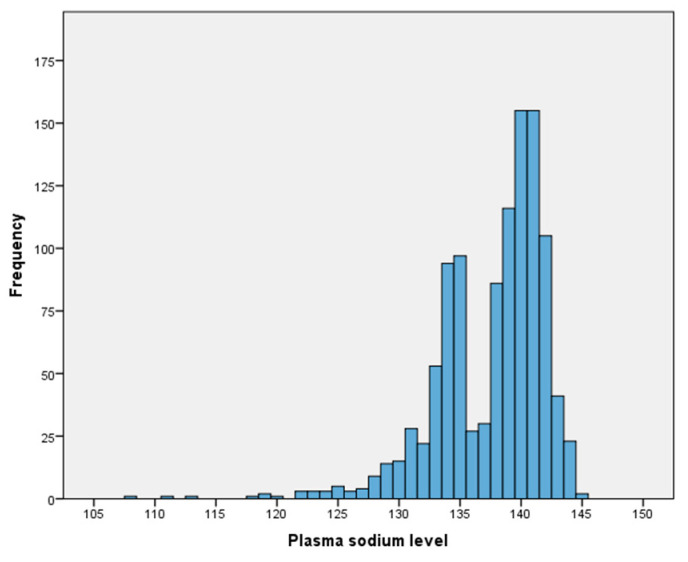
The distribution of sodium levels in the study population.

The mean age of the 1100 participants was 75.59 ± 8.13 years, and 64% were female. Demographic characteristics, comorbidities, drugs, and laboratory data of the patients with and without hyponatremia are summarized in Table 1. The prevalence of hyponatremia was 23.9%. The distribution of sodium levels in the study population is presented in Figure 2. The patients with hyponatremia were older than those with normonatremia (*p* < 0.001). In the hyponatremia group, the rates of dementia, falls, polypharmacy, malnutrition and risk of malnutrition (MRM), probable sarcopenia, and frailty were higher, whereas Basic and Instrumental ADLs, MNA-SF, and POMA scores were worse, than in the normonatremia group, all of which are shown in Table 2 in detail. The presence of dementia, falls, polypharmacy, frailty, MRM, low muscle strength, and usage of hydrochlorothiazide, furosemide, escitalopram, trazodone, opioids, PPIs, and RAAS inhibitors were associated with hyponatremia (*p* < 0.05). After adjustment for age, sex, the total number of drugs (except for polypharmacy), the Charlson Comorbidity Index, glucose and albumin levels, and eGFR, hyponatremia was associated with the use of escitalopram (odds ratio [OR]: 1.82, 95% confidence interval [CI]: 1.20–2.76), trazodone (OR: 2.27, 95% CI: 1.26–4.10), renin–angiotensin–aldosterone system (RAAS) inhibitors (OR: 1.71, 95% CI: 1.18–2.47), hydrochlorothiazide (OR: 1.83, 95% CI: 1.28–2.62), opioids (OR: 4.46, 95% CI: 1.24–16.02), falls (OR: 1.45, 95% CI: 1.02–2.05), polypharmacy (OR: 1.86, 95% CI: 1.27–2.73), and MRM (OR: 2.46, 95% CI: 1.66–3.65) (*p* < 0.05). The associations between drugs, geriatric syndromes, and hyponatremia are summarized in Table 3.

## 4. Discussion

This study shows that hyponatremia is linked to the use of hydrochlorothiazide, trazodone, escitalopram, opioids, and RAAS inhibitors. Additionally, hyponatremia is associated with geriatric syndromes, including malnutrition and risk of malnutrition, and polypharmacy, as well as falls independent of age and comorbidities. It is worth noting that no relationship was found between hyponatremia and commonly blamed medications like indapamide, SNRIs, and antipsychotics.

It was reported that up to one-third of older adults had serum sodium <135 mmol/L [20]. In the Rotterdam Study, a population-based study, the prevalence of hyponatremia was found to be 11.6% among individuals aged 75 years or older [21], and the severity and prevalence increased with age [22,23]. The NHANES study also showed that the frequency of hyponatremia was more common in women, older adults, and those with comorbidities, such as hypertension, diabetes, coronary artery disease, stroke, chronic obstructive pulmonary disease, cancer, and psychiatric disorders [24]. In accordance with the literature, we found that patients with hyponatremia had more frequent hypertension, congestive heart failure (CHF), cerebrovascular disease (CVD), and dementia and were older than those with normonatremia. In addition, the risk of hyponatremia was increased with geriatric syndromes, including sarcopenia, frailty, falls, malnutrition, dementia, and polypharmacy in the present study. Compelling evidence suggested that hyponatremia was a potential marker of unsuccessful aging or poor physiological body reserve. In addition, hyponatremia is one of the most common reasons for older adults to be admitted to the emergency department [25,26]. A cohort study showed that older patients (≥75 years) with symptomatic moderate to severe hyponatremia who had recurrent hospitalizations due to hyponatremia had higher one-year mortality than those who had a single hospitalization due to hyponatremia (42.5% vs. 23.8%; *p* < 0.0001) [27]. Furthermore, it was also reported that older patients with mild-to-moderate hyponatremia displayed worse performances in cognitive and functional tests [28]. Accordingly, in the present study, the hyponatremia group had worse scores in mobility, functionality, and nutrition, as well as frailty. However, after the adjustment of confounders, hyponatremia was only related to polypharmacy, falls, and malnutrition. In such conditions, increased intravenous or oral fluid intake and non-osmotic ADH secretion that may cause hypo-osmolality may be major potential mechanisms leading to hyponatremia [3,29]. Furthermore, in inpatients, hyponatremia may result from various factors, such as pain, nausea, postsurgical stress, and medications that cause hyponatremia [8,30]. In such cases, the kidneys may be unable to eliminate excess water, leading to or worsening hyponatremia. Patients who are malnourished and have inflammation-associated diseases may have elevated levels of proinflammatory cytokines like IL-1 Beta and IL-6, which can trigger non-osmotic ADH secretion and exacerbate hyponatremia [31]. Furthermore, hyponatremia was reported as an essential predictor of falls, for which the potential confounders reported were female gender, advanced age, functional limitation, comorbidities, especially dementia syndromes and neurological disorders (dementia, history of stroke, Alzheimer’s disease, Parkinson’s disease), and cardiovascular disease [32].

The present study showed that the number of drugs in hyponatremic patients was higher than in the other patients, as reported in the previous studies [7,28,33]. The most commonly blamed drugs for hyponatremia are RAAS inhibitors, antipsychotics, antidepressants, anticonvulsants, diuretics, and PPIs [1,7,10,34,35,36,37,38]. Moreover, given the frequent prescription of such drugs to older adults, the importance of drug-related hyponatremia may be better understood for geriatric practice. Among these drugs, RAAS inhibitors were found to be associated with hyponatremia in our study, as reported in previous studies [34,39]. The mechanisms of RAAS inhibitor (ACEIs and ARBs)-related hyponatremia may involve reduced sodium reabsorption due to the down-regulation of epithelial sodium channels in the distal nephron, particularly in the distal convoluted tubule, connecting tubule, and cortical collecting duct, the suppression of renin expression in renal tubular cells despite a compensatory increase in systemic renin, and the inhibition of angiotensin II-mediated aldosterone secretion [34,40]. Besides these potential mechanisms, ACEI may cause SIADH, which needs to be clarified. However, experimental studies reported that ACEIs in low to moderate doses block the conversion of angiotensin-I (AT-I) to angiotensin-II (AT-II) in the peripheral circulation but not in the brain. So, AT-I turns into AT-II in the brain, increasing the feeling of thirst and ADH secretion [37]. In addition, it should be noted that older adults with hypertension are often on combination therapy, including thiazides and RAAS inhibitors, which augments the hyponatremic effects of RAAS inhibitors. Accordingly, our findings showed that the use of hydrochlorothiazide is related to an increased risk of hyponatremia, independent of age and comorbidities. On the other hand, the use of low-dose indapamide has been reported to be likely to cause severe hyponatremia in older adults [41,42]; however, we were unable to find any association between indapamide and hyponatremia in the study population, which may be related to the low number of patients receiving indapamide in older adults. Antidepressants are another drug group that may lead to hyponatremia in older adults. Selective serotonin reuptake inhibitors (SSRIs), a particular group of antidepressants, are more prevalent in this condition, which may be frequently related to the inappropriate release of ADH [43]. Among SSRIs, it was remarked that unlike paroxetine and sertraline, escitalopram, citalopram, and fluoxetine are more likely to be related to hyponatremia [43,44,45,46], in accordance with our results. In addition, trazodone was also found to be associated with mild hyponatremia and to increase the risk of hyponatremia by nearly 2.3 times, even at low doses (25–100 mg/day), in our study population. However, trazodone-associated hyponatremia has been reported in only a few case reports with toxic doses of the drug in older adults [47] and in a retrospective study conducted with unselected young psychiatric inpatients [48] so far. Therefore, to the best of our knowledge, our study is the first one to demonstrate the association between hyponatremia and trazodone, even at lower doses, in older adults. Since trazodone, a safer and better-tolerated serotonin antagonist and reuptake inhibitor [49], is considered to be a preferred drug in older adults with insomnia due to its less likely adverse effects profile at the low doses [50], older adults treated with trazodone should be monitored for the development of hyponatremia secondary to SIADH, similar to SSRIs. Moreover, this study demonstrated no relationship between antipsychotic medication and hyponatremia, which may be attributed to our high awareness of the anticholinergic side effects as a referral memory center and the limited prescription of these medications in our practice. Furthermore, opioids, which are commonly administered medication in geriatric practice, may also be associated with hyponatremia in older adults by inhibiting the central reuptake of serotonin and norepinephrine. On the other hand, pain itself may result in hyponatremia owing to the secretion of non-osmotic ADH. Tramadol, an opioid agonist, is reported to be linked to an increased risk of hospitalization due to hyponatremia [51]. Concurrently, opioids were shown to increase the risk of hyponatremia by almost 4.5 times in this study.

This study has several strengths, including a large sample size and a detailed review of different drug classes. All participants underwent a comprehensive geriatric assessment, and eight major geriatric syndromes were screened. Additionally, this study examines the relationship between geriatric syndromes and hyponatremia using sophisticated statistical analysis. However, there are also limitations. The retrospective design lacks follow-up data, limiting long-term outcome assessment. While we analyzed the individual effects of RAAS inhibitors, diuretics, and antidepressants, their cumulative effects were not specifically evaluated. Furthermore, the volemic status was not systematically assessed due to the retrospective nature of this study. Future prospective studies incorporating detailed volemic evaluations and analyzing the combined impact of multiple medications would provide deeper insights into the mechanisms of hyponatremia in older adults.

## 5. Conclusions

This study demonstrated that hyponatremia appears to be associated with certain geriatric syndromes, as well as the use of some antidepressants, tramadol, and cardiovascular drugs in older adults. In addition, older adults, especially malnourished ones, taking RAAS inhibitors, diuretics, and antidepressants may be at a higher risk of developing hyponatremia. Hence, they should be closely monitored, especially if taking multiple medications. These findings suggest that the causes of hyponatremia may be more complex than previously thought. Further studies are needed to understand the exact mechanisms and factors related to hyponatremia development and to interpret the outcomes in older patients.

## Figures and Tables

**Table 1:** Demographic characteristics and comorbidities of the patients with and without hyponatremia.

	Normonatremia *N* = 837	Hyponatremia *N* = 263	*p*-Value
**Demographic Features (%)**			
Age (years)	74.62 ± 7.77	78.69 ± 8.48	<0.001
Sex (female)	63.2	66.9	0.275
Education (years)	7.54 ± 4.65	6.62 ± 4.69	0.006
Marital Status (married)	58.5	49.2	0.075
Number of Drugs Used	5.27 ± 3.17	7.07 ± 3.56	<0.001
Length of Stay	5.41 ± 2.67	6.37 ± 3.57	0.038
**Comorbidities (%)**			
Hypertension	64.8	78.4	<0.001
Diabetes Mellitus	27.7	33.5	0.075
Atherosclerotic Coronary Artery Disease	19.3	20.8	0.591
Hyperlipidemia	18.0	21.3	0.228
Congestive Heart Failure	6.3	11.6	0.004
Cerebrovascular Disease	6.7	10.5	0.045
Chronic Obstructive Pulmonary Disease	8.9	11.3	0.250
Dementia	24.7	34.3	0.003
Charlson Comorbidity Index	1.26 ± 1.39	1.60 ± 1.44	<0.001
**Drugs (%)**			
Hydrochlorothiazide	28.0	42.2	<0.001
Furosemide	5.9	12.5	<0.001
Indapamide	3.9	6.5	0.087
SSRI	29.8	37.6	0.019
Trazodone	5.4	12.8	<0.001
PPIs	20.5	33.8	<0.001
RAAS Inhibitors	49.6	63.9	<0.001
Antipsychotics	10.3	12.9	0.655
Opioids	0.7	3.1	0.007
**Laboratory Findings (Mean ± SD)**			
Glucose	109.97 ± 39.91	123.48 ± 65.36	0.007
eGFR (mL/min per 1.73 m^2^)	74.83 ± 18.85	69.68 ± 23.61	0.002
Albumin (g/dL)	4.03 ± 0.36	3.78 ± 0.53	<0.001
TSH (mIU/L)	1.84 ± 2.35	2.24 ± 7.19	0.468

eGFR: estimated glomerular filtration rate, RAAS: renin–angiotensin–aldosterone system, PPIs: proton pump inhibitors, SD: standard deviation, TSH: Thyroid-Stimulating Hormone.

**Table 2:** Patients’ comprehensive geriatric assessment parameter scores and geriatric syndrome frequencies.

	Normonatremia *N* = 837	Hyponatremia *N* = 263	*p*-Value
**Comprehensive Geriatric Assessment Parameters** **(Mean ± SD)**			
MMSE	23.33 ± 6.63	22.73 ± 6.04	0.061
YGDS	3.20 ± 3.35	3.47 ± 3.40	0.235
Basic ADLs	89.11 ± 15.88	73.47 ± 28.42	<0.001
Instrumental ADLs	17.09 ± 6.49	11.68 ± 8.01	<0.001
POMA total	24.50 ± 5.01	19.75 ± 8.29	<0.001
Up and Go (sec)	13.92 ± 7.96	19.03 ± 11.91	<0.001
MNA-SF	12.13 ± 2.26	10.36 ± 3.29	<0.001
**Geriatric Syndromes (%)**			
Falls	29.8	44.4	<0.001
Urinary Incontinence	50.4	52.1	0.632
Dementia	24.7	34.3	0.003
Depression	33.2	33.2	0.993
Polypharmacy	54.8	74.0	<0.001
Malnutrition and Risk of Malnutrition	27.5	52.9	<0.001
Probable sarcopenia	59.8	72.3	0.016
Frailty	26.0	45.5	<0.001

Abbreviations: MMSE: Mini-Mental State Examination (0–30), YGDS: Yesavage Geriatric Depression Scale (0–15), ADL: Activities of Daily Living, POMA: Performance-Oriented Mobility Assessment (0–28), MNA-SF: Mini-Nutritional Assessment Short Form (0–12).

**Table 3:** The association between hyponatremia, drug, and geriatric syndromes.

	Model 1 *			Model 2 **		
	Odds Ratio (Beta)	Confidence Interval (CI) %95	*p*-Value	Odds Ratio (Beta)	Confidence Interval (CI) %95	*p*-Value
**Diuretics**						
Hydrochlorothiazide	1.88 (0.63)	1.41–2.51	<0.01	1.83 (0.60)	1.28–2.62	<0.01
Furosemide	2.31 (0.84)	1.45–3.67	<0.01	0.94 (−0.07)	0.51–1.73	0.83
Indapamide	1.68 (0.52)	0.92–3.08	0.09	1.07 (−0.06)	0.51–2.24	0.87
**Antidepressants**						
Escitalopram	1.76 (0.57)	1.24–2.50	<0.01	1.82 (0.60)	1.20–2.76	<0.01
SNRIs	0.83 (−0.19)	0.39–1.74	0.62	0.88 (−0.13)	0.38–2.04	0.76
Trazodone	2.55 (0.94)	1.59–4.10	<0.01	2.27 (0.82)	1.26–4.10	<0.01
**PPIs**	1.98 (0.68)	1.46–2.68	<0.01	1.36 (0.31)	0.93–2.00	0.11
**RAAS inhibitors**	1.80 (0.59)	1.35–2.39	<0.01	1.71 (0.54)	1.18–2.47	<0.01
**Antipsychotics**	1.13 (0.12)	0.94–1.37	0.17	0.66 (−0.42)	0.38–1.15	0.14
**Opioids**	4.22 (1.44)	1.51–11.77	<0.01	4.46 (1.49)	1.24–16.02	0.02
**NSAIDs**	1.14 (0.13)	0.69–1.88	0.59	1.02 (0.02)	0.53–1.96	0.96
**Dementia**	1.58 (0.46)	1.17–2.14	<0.01	1.03 (0.03)	0.70–1.52	0.87
**Depression**	1.00 (0.00)	0.74–1.35	0.99	0.85 (−0.16)	0.60–1.22	0.39
**Falls**	1.87 (0.63)	1.41–2.50	<0.01	1.45 (0.37)	1.02–2.05	0.04
**Polypharmacy**	2.35 (0.85)	1.72–3.20	<0.01	1.86 (0.62)	1.27–2.73	<0.01
**Frailty**	2.36 (0.86)	1.53–3.64	<0.01	1.54 (0.43)	0.90–2.63	0.11
**Malnutrition and Malnutrition risk**	2.96 (1.08)	2.16–4.04	<0.01	2.46 (0.90)	1.66–3.65	<0.01
**Probable sarcopenia (low muscle strength)**	1.75 (0.56)	1.10–2.78	0.01	1.28 (0.25)	0.74–2.22	0.37

Abbreviations: SNRIs: serotonin and norepinephrine reuptake inhibitors, PPIs: proton pump inhibitors, RAAS: renin–angiotensin–aldosterone system, NSAIDs: non-steroidal anti-inflammatory drugs. * Model 1 was unadjusted. ** Model 2 was adjusted for age, sex, total drug number (except for polypharmacy), Charlson comorbidity index, glucose, albumin, and eGFR (estimated glomerular filtration rate).

## Data Availability

The datasets used and analyzed during the present study are available from the corresponding author upon reasonable request.
